# Exposure to climate change information predicts public support for solar geoengineering in Singapore and the United States

**DOI:** 10.1038/s41598-023-46952-w

**Published:** 2023-11-14

**Authors:** Sonny Rosenthal, Peter J. Irvine, Christopher L. Cummings, Shirley S. Ho

**Affiliations:** 1 Wee Kim Wee School of Communication and Information, Nanyang Technological University, 1 Nanyang Link #03-48, Singapore, 637718 Singapore; 2 UCL Earth Sciences, University College London, London, UK; 3 Genetic Engineering and Society Center, North Carolina State University, Raleigh, USA

**Keywords:** Climate-change mitigation, Climate-change policy, Psychology and behaviour

## Abstract

Solar geoengineering is a controversial climate policy measure that could lower global temperature by increasing the amount of light reflected by the Earth. As scientists and policymakers increasingly consider this idea, an understanding of the level and drivers of public support for its research and potential deployment will be key. This study focuses on the role of climate change information in public support for research and deployment of stratospheric aerosol injection (SAI) in Singapore (*n* = 503) and the United States (*n* = 505). Findings were consistent with the idea that exposure to information underlies support for research and deployment. That finding was stronger in the United States, where climate change is a more contentious issue, than in Singapore. Cost concern was negatively related to support for funding and perceived risk was negatively related to support for deployment. Perceived government efficacy was a more positive predictor of support for funding in Singapore than in the United States. Additionally, relatively low support for local deployment was consistent with a NIMBY mindset. This was the first study to quantify the role of climate change information in SAI policy support, which has practical implications for using the media and interpersonal channels to communicate about SAI policy measures.

## Introduction

Solar geoengineering, also called solar radiation management, is a proposal to lower global temperatures by reducing the amount of light absorbed by the Earth. One approach is to release tiny particles high in the atmosphere to reflect sunlight into space, mimicking the natural cooling effect of sulfur-rich volcanic eruptions^1^. Scientists agree that this approach, called stratospheric aerosol injection (SAI), could be effective at slowing global warming within years^2–4^. However, there are concerns it could deplete stratospheric ozone, contribute to acid rain, and create a moral hazard by drawing interest and resources away from mitigation^4,5^. The United Nations report, *One Atmosphere*^3^, highlighted these issues.

Public opinion about SAI can provide additional insights about its development and deployment. Political leaders are often responsive to public demands to address climate change^6,7^, and there have been calls from policymakers^8^ and scholars^9^ for more upstream public engagement about solar geoengineering, especially its governance^10,11^. Most people have low knowledge about SAI and often express concern upon learning about it^12^. Mercer et al.^13^ noted that public opinion research is needed to effectively deal with future climate change policy. Their cross-national survey showed that public support for solar geoengineering was positively related to the beliefs that it will be effective, easy to implement, and safe. Asayama et al.^14^ found that people were more accepting of SAI field trials framed as a solution to climate change but had concerns over accountability and controllability. Other research showed that the public desires to understand the efficacy, costs, and risks of SAI and other climate engineering approaches^15,16^.

The points above align with secondary risk theory^17^, an extension of protection motivation theory^18^ stating that people favor risk reduction interventions they perceive to be implementable and effective, while opposing those they believe are too expensive or risky to deploy. Scholars have used that theory in the contexts of pandemic response and vaccine hesitancy^19–21^, terrorism countermeasures^22^, critical infrastructure risk assessment^23^, and urban development and vulnerability^24^, and it could be usefully applied in the context of SAI. If the public believes climate change will harm them and SAI can be effectively deployed to reduce that threat, then they will tend to support its deployment. In contrast, if they believe SAI would draw money away from other important projects or have harmful side effects, they will be less likely to support it. One aim of this study is to test those drivers of public support.

Another aim of this study is to understand the role of information in the formation of beliefs about climate change and SAI. Members of the lay public do not deal with these issues directly in their everyday lives; rather, they depend on the media and interpersonal conversations to gain perspectives they can use to form opinions^25,26^. More than a decade ago, Mercer et al.^13^ noted increasing news coverage of geoengineering. Recent evidence has shown that news consumption is positively related to familiarity with geoengineering^27^ and exposure to information about it can trigger affective-cognitive evaluations related to perceived risk^28^. At least in the United States, increasing media coverage of SAI has emphasized its negative aspects, and such negative framing reduces public support for SAI research and deployment^29,30^. The following subsections review the literature on secondary risk theory and risk communication, which we use to develop several hypotheses.

### Secondary risk theory

People may respond to environmental and health risks by engaging in protective behaviors, or *risk response actions*. According to protection motivation theory^18^, individuals are motivated to engage such actions when they perceive they are vulnerable to a severe risk—termed a *threat appraisal*—and feel capable of enacting an effective action to reduce or avoid the risk—termed a *coping appraisal*. According to the theory, individuals must experience high levels of both threat appraisal and coping appraisal to feel motivated to act. They will be unmotivated if they feel that they are not vulnerable to the risk, the risk is mild, they are unable to perform a protective behavior, or the protective behavior will be ineffective. Also, individuals will be less motivated to engage in risk response actions they find costly in terms of time, effort, money, or another valued object. Meta-analyses and systematic reviews have found support for this model in many behavioral contexts, including health^31^, information security^32^, and the environment^33^.

In some instances, a risk response action can create its own real or perceived risks^34^. In the case of SAI, the public may be unsupportive of funding or deploying it if they perceive it as risky to their health or the environment^28^. Believing a risk response action poses a severe and likely risk constitutes a *secondary threat appraisal*, which secondary risk theory^17,35^ includes as an additional predictor of protection motivation. This parallels response costs, which alone may fail to explain opposition to risky risk response actions^36^. Accordingly, individuals are motivated to engage in risk response actions when there is a high primary threat appraisal and coping appraisal, and demotivated when there are high perceived costs or there is a high secondary threat appraisal.

When risk response actions involve technological solutions that require action at an institutional or societal level, rather than an individual level, it makes sense that coping appraisal and perceived cost would reflect that societal-level factor^37,38^. In the case of response efficacy, individuals generally cannot deploy SAI on their own and depend on governments to pursue funding and deployment. Studies have shown that confidence in the government to deal with environmental problems is positively related to support for environmental policies in the contexts of environmental protection^39^ and climate change adaptation^40^. In the case of response costs, funding and deploying SAI will likely require government expenditure, posing an indirect cost to taxpayers that may reduce funding for other important projects. Such perceived costs are important because when they are high, individuals are less supportive of climate policies and less sensitive to other factors, such as urgency, that might otherwise motivate their policy support^41^. We propose to replicate secondary risk theory in this novel context:

#### Hypothesis 1

The perceived risk of climate change is positively related to support for (a) funding SAI research and (b) SAI deployment.

#### Hypothesis 2

The perceived efficacy of SAI is positively related to support for (a) funding SAI research and (b) SAI deployment.

#### Hypothesis 3

The perceived efficacy of the government to deal with climate change is positively related to support for (a) funding SAI research and (b) SAI deployment.

#### Hypothesis 4

The perceived financial cost of SAI is negatively related to support for (a) funding SAI research and (b) SAI deployment.

#### Hypothesis 5

The perceived risk of SAI is negatively related to support for (a) funding SAI research and (b) SAI deployment.

### Deference to scientific authority

Studying risk response actions, particularly those related to environmental and health risks, can benefit from contextualization vis-à-vis the public’s scientific beliefs. Researchers have used deference of scientific authority alongside threat appraisal and coping appraisal to predict acceptance of nanofood technology^42^ and support for releasing genetically modified mosquitoes^43^. Those studies found that individuals with a greater deference to scientific authority were more supportive of those technologies. Scholars have defined deference to scientific authority as a lasting disposition reflecting the belief that “decision-making concerning science and technology should be the purview of the scientific community and not part of larger democratic discourse”^44^. It puts technocracy above democracy, pragmatism above humanism^45^. This concept has clear import to models of public support for technological solutions to climate change, such as SAI.

#### Hypothesis 6

Deference to scientific authority is positively related to support for (a) funding SAI research and (b) SAI deployment.

### Exposure to information

Because climate change is a subtle phenomenon, people depend on second-hand information to learn about it^26,46^. The belief that climate change will have harmful consequences often comes from reading news and government reports, watching films and documentaries, and having conversations with laypersons and experts alike^25,47^. Likewise, technological solutions to climate change, such as SAI, involve physical and chemical processes that the public generally cannot observe directly. Therefore, individuals depend on information sources to form beliefs about the benefits and risks of such solutions^27,28^.

Risk-related information can affect how people understand the characteristics of a risk and their options for dealing with it. Guo et al.^48^ found that exposure to typhoon-related information from traditional and new media sources predicted typhoon disaster preparedness and emergency responses. Witzling et al.^49^ found that boaters exposed to information about aquatic invasive species had more knowledge about and more positive attitudes toward measures to prevent the spread of invasive species, which predicted their compliance with those measures. The findings of both studies are consistent with the idea that exposure to environmental information influences the cognitive determinants of environmental behaviors.

In the context of climate change, individuals can use the media and other information sources to learn about its potential impacts, which may affect their support for climate change solutions. An analysis of climate change in the media found frequent images of catastrophe and visualizations of greenhouse gas emissions and warming trends^50^. Feldman and Hart^51^ showed that individuals exposed to information about the negative impacts of climate change expressed higher levels of fear and were more supportive of mitigation policies. Those mitigation policies included measures to reduce carbon dioxide emission through the regulation of emitters, subsidies of renewables, and international agreements. We expect such a process would also explain support for SAI.

Whereas it is straightforward that exposure to information about climate change could affect beliefs about climate change, we also argue that exposure to information about climate change could affect beliefs about SAI. The reason is that climate change information provides a basis for individuals to gauge the desirability of mitigation approaches. Individuals exposed to climate change information may be more supportive of activities that slow the warming process and help avert catastrophe, as long as they are aware of available actions. Feldman and Hart^51^ showed that individuals expressed more hope when they were exposed to information about both the negative impacts of climate change and possible actions to address it. In turn, hope predicted their support for mitigation policies. In the case of SAI, individuals who are aware of it as a potential solution may feel more hopeful about its efficacy and express more support for it when they have encountered more information about climate change.

**Figure 1:**
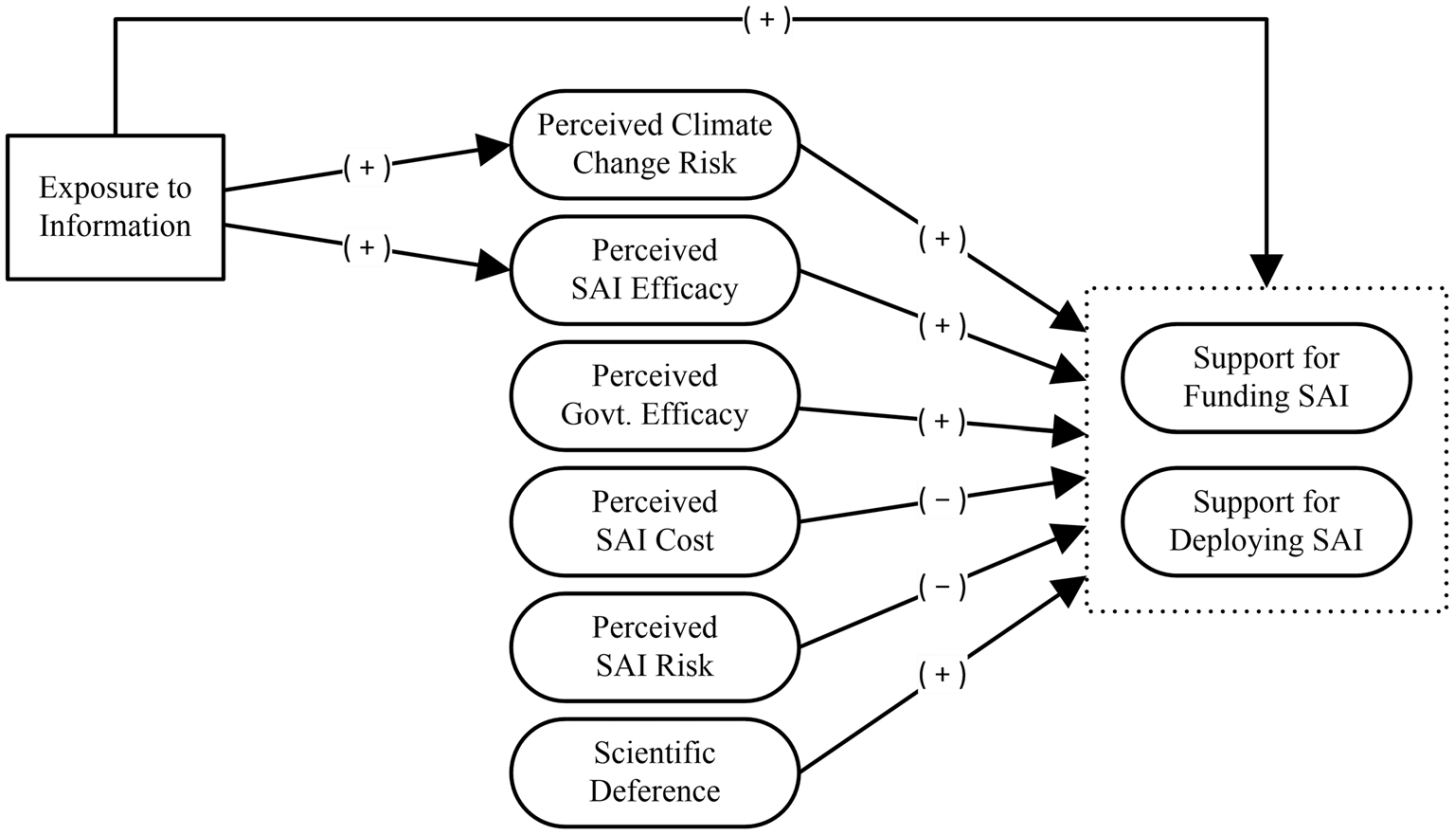
Mediation model of support for SAI. Corresponding with our statistical model (see Methods), the solid rectangle reflects an observed variable and ovals represent latent variables. For visual simplicity, we have grouped the two types of support within the dotted rectangle. This figure omits the control variables.

Based on secondary risk theory and scholarship on risk communication, we propose a mediation model in which exposure to information is indirectly related to support for SAI funding and deployment via beliefs about climate change and SAI (see Fig. 1).

#### Hypothesis 7

Exposure to climate change information positively predicts support for (a) funding SAI research and (b) SAI deployment.

#### Hypothesis 8

The relationship between exposure to information and support for funding SAI research is positively mediated by the (a) the perceived risk of climate change and (b) the perceived efficacy of SAI.

#### Hypothesis 9

The relationship between exposure to information and support for SAI deployment is positively mediated by the (a) the perceived risk of climate change and (b) the perceived efficacy of SAI.

### National context

Public opinion about SAI also differs between countries. Visschers et al.^52^ found that support for SAI was the highest in China and lowest in Canada and the United States. Germany, Switzerland, and the United Kingdom were in between. Similarly, Sugiyama et al.^53^ reported that support for SAI was higher in the Global South than in the Global North. Within the latter group, there was weaker support in Japan and South Korea than in Australia, hinting at an East–West divide. The current study provides another data point using public opinion data from Singapore and the United States. This allows us to address a general research question, does public support for SAI and its prediction differ between Singapore and the United States?

One way that Singapore and the United States appear to differ is how their publics engage with climate policies. There is evidence of a distinct worldview in the United States that affects appraisals of climate change^54^. In a related context, Liang et al.^55^ found that deference to scientific authority positively predicted support for nanotechnology research, which was stronger in the United States than in Singapore. In contrast with the United States, Singapore is known for broad public approval of a strong central government^56,57^. It is also known for its technocratic approach to environmental governance^58^. Thus, it makes sense that policy support in Singapore will be more influenced by the public’s beliefs about the government than by their beliefs about the policy’s scientific merits. This will be the opposite in the United States.

#### Hypothesis 10

Deference to scientific authority is more positively related to support for (a) funding SAI research and (b) SAI deployment in the United States than in Singapore.

#### Hypothesis 11

Perceived government efficacy is more positively related to support for (a) funding SAI research and (b) SAI deployment in Singapore than in the United States.

Also, whereas freedom of speech is entrenched in the United States, there is considerable regulation of speech in Singapore. For instance, in 2019, the Singapore Parliament enacted a law enabling ministers to issue takedown notices to anyone who publishes online messages they have determined are false. This law could be used to combat misinformation about climate change^59^ and, coupled with the government’s approach to environmental policymaking, may create a unique environment for the Singapore public to engage with information about climate change^57^ and form opinions about related issues like SAI. In contrast, the relatively unbridled information landscape in the United States means the public may be exposed to a wider range of viewpoints—including those that go against scientific consensus—which could result in more varied opinions. Consistent with that argument, segmentation studies have shown that public opinion on climate change is more diverse in the United States than in Singapore^60,61^.

#### Hypothesis 12

Exposure to information is more strongly related to (a) the perceived risk of climate change and (b) perceived efficacy of SAI in the United States than in Singapore.

#### Hypothesis 13

The indirect effects of exposure to information on support for funding SAI research via (a) the perceived risk of climate change and (b) the perceived efficacy of SAI are more positive in the United States than in Singapore.

#### Hypothesis 14

The indirect effects of exposure to information on support for SAI deployment via (a) the perceived risk of climate change and (b) the perceived efficacy of SAI are more positive in the United States than in Singapore.

## Results

Data came from an online survey of 1008 adults in Singapore (*n* = 503) and the United States (*n* = 505) who read a short description of SAI (see supplementary information) and responded to items measuring their support for government funding and deployment. Control variables in our regression models included age, education, political orientation, and communitarianism, which can explain public support for climate policies^62,63^. Focal predictors included the perceived risk of climate change; perceived government efficacy to address climate change; the perceived efficacy, financial cost, and risk of SAI; exposure to information; and deference to scientific authority (see Table S1 for item wordings).

### Descriptive summary

Respondents reported the highest support for funding SAI research (*M* = 3.48, *SD* = 1.03) followed by funding development (*M* = 3.33, *SD* = 1.03), regional deployment (*M* = 3.24, *SD* = 1.04), and local deployment (*M* = 3.18, *SD* = 1.04). All those values were different from each other (*p*s < 0.017) and higher than the neutral scale midpoint (*p*s < 0.001). That latter finding means that respondents tended to express support for SAI research and deployment. Support for funding development was higher in Singapore than the United States (Δ*M* = 0.16, *p* = 0.016). Otherwise, support was not different between countries. Figure S1 (supplementary information) shows the frequency of responses to the four items measuring support for SAI.

Looking at the predictor variables, the Singapore sample reported higher communitarianism (Δ*M* = 0.26, *p* < 0.001), perceived risk of climate change (Δ*M* = 0.30, *p* < 0.001), and perceived government efficacy (Δ*M* = 0.35, *p* < 0.001). There were no other differences in mean scores between countries.

### Main effects

To replicate secondary risk theory, we estimated a regression model predicting support for SAI. In the pooled sample, support for funding was positively related to perceived SAI efficacy (β = 0.57, *p* < 0.001) and deference to scientific authority (β = 0.37, *p* < 0.001). The total effect of exposure to information on support for funding, which included the direct and indirect effects, was positive (β = 0.22, *p* < 0.001). These findings supported hypotheses 2a, 6a, and 7a. The model explained 68% of the variance in support for funding (see Table 1).

**Table 1:**
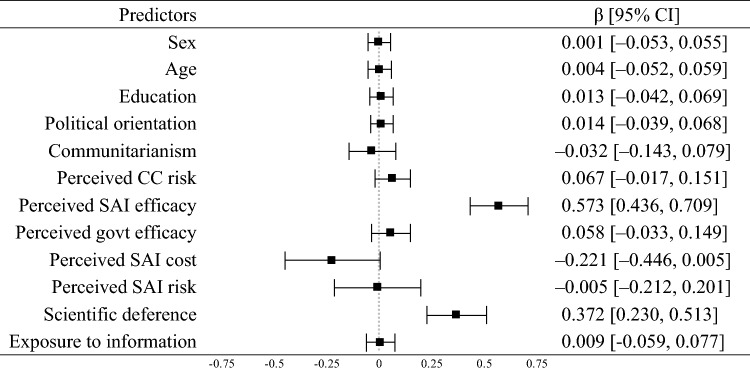
Linear regression of support for SAI funding (pooled sample; *R*^2^ = 0.68).

*CC* climate change, *SAI* stratospheric aerosol injection, Squares show the standardized regression point estimates (β) with error bars showing the 95% confidence intervals (CI). Confidence intervals were estimated with 5000 bias-corrected bootstrap samples.

Support for deployment was positively related to perceived climate change risk (β = 0.09, *p* = 0.025), perceived SAI efficacy (β = 0.57, *p* < 0.001), perceived government efficacy (β = 0.09, *p* = 0.039), and deference to scientific authority (β = 0.24, *p* < 0.001). It was negatively related to perceived SAI risk (β = -0.32, *p* < 0.001). The total effect of exposure to information on support for deployment, which included the direct and indirect effects, was positive (β = 0.27, *p* < 0.001). These findings supported hypotheses 1b, 2b, 3b, 5b, 6b, and 7b. The model explained 69% of the variance in support for deployment (see Table 2). Overall, these findings provided mixed support for secondary risk theory.

**Table 2:**
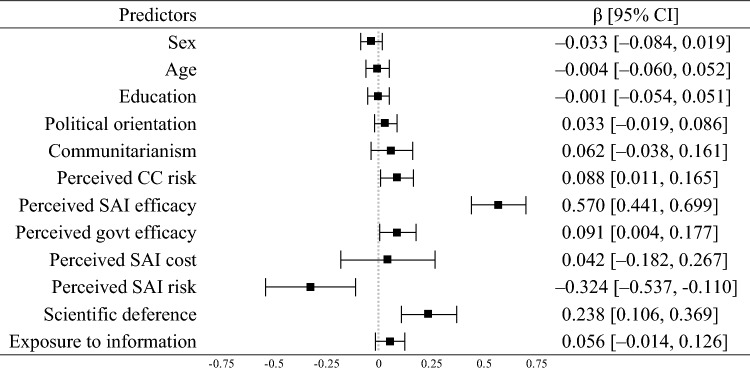
Linear regression of SAI deployment (pooled sample; *R*^2^ = 0.69).

*CC* climate change, *SAI* stratospheric aerosol injection. Squares show the standardized regression point estimates (β) with error bars showing the 95% confidence intervals (CI). Confidence intervals were estimated with 5,000 bias-corrected bootstrap samples.

### Indirect effects

To test indirect effects, first we established that exposure to information was positively related to perceived climate change risk (β = 0.27, *p* < 0.001) and perceived SAI efficacy (β = 0.33, *p* < 0.001). Next, we examined a mediation model consistent with the argument that support for SAI is related to exposure to climate change information because information affects beliefs about climate change and SAI. Consistent with hypothesis 8b, support for funding was indirectly related to exposure to information via perceived SAI efficacy (β = 0.19, 95% CI [0.12, 0.26]). Consistent with hypotheses 9a and 9b, support for deployment was indirectly related to exposure to information via perceived climate change risk (β = 0.02, 95% CI [0.001, 0.05]) and perceived SAI efficacy (β = 0.19, 95% CI [0.12, 0.25]). See Table 3.

**Table 3:**
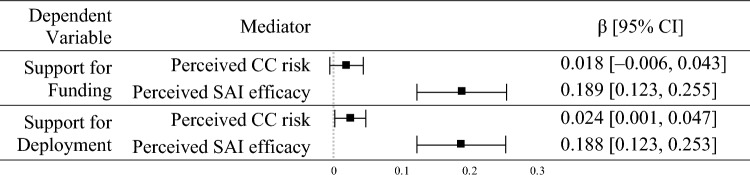
Indirect effects of exposure to information on support for funding and deployment.

This shows the effect of exposure to information → mediator → support for SAI, where each line corresponds to a different mediator. *CC* climate change, *SAI* stratospheric aerosol injection. Squares show the standardized regression point estimates (β) with error bars showing the 95% confidence intervals (CI). Confidence intervals were estimated with 5,000 bias-corrected bootstrap samples.

### Between-country differences

A significant chi-square difference test, Δχ^2^(51) = 99.14, *p* < 0.001 (see Table S2 note), indicated the regression model was different between countries. Multigroup analysis showed the relationship between government efficacy and support for funding was more positive in Singapore than in the United States, (*p* = 0.034), which supported hypothesis 10a. Otherwise, there were no between-country differences in the prediction of support for funding (see Table S3) or deployment (see Table S4).

Multigroup analysis showed that exposure to information was more positively related to perceived SAI risk (*p* = 0.01) and perceived SAI efficacy (*p* = 0.008) in the United States than in Singapore (see Table S4). These findings support hypotheses 12a and 12b. In the United States, there was a more positive indirect effect of exposure to information via perceived SAI efficacy on support for SAI funding (*p* = 0.006) and deployment (*p* = 0.038). These findings supported hypotheses 13b and 14b (see Table 4).

**Table 4:**
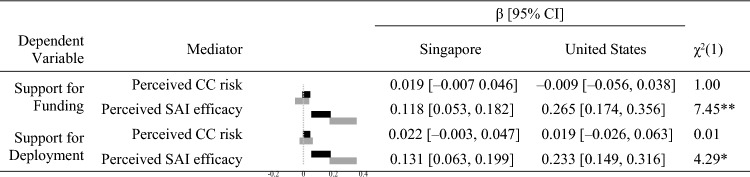
Indirect effects of exposure to information on support for funding in Singapore and the United States.

This shows the effect of exposure to information → mediator → support for SAI, where each line corresponds to a different mediator. *CC* climate change, *SAI* stratospheric aerosol injection, Bars show the 95% confidence intervals (CI) of the standardized regression paths (β) in Singapore (black) and the United States (gray). Confidence intervals were estimated with 5000 bias-corrected bootstrap samples. χ^2^(1) is the chi-square test with one degree of freedom comparing each regression path between countries.

## Discussion

As scientists continue to study SAI and policymakers increasingly consider its use, there is a need for social science research to understand the levels and drivers of public support for its research and potential deployment. This study showed in Singapore and the United States higher levels of support for funding research than for deployment. This is consistent with Mercer, et al.^13^, who found public support was the highest for SAI research, lower for emergency deployment, and the lowest for immediate deployment. Our analysis added two nuances. First, there was more support for funding research than for funding development. Assuming that respondents interpreted “research” to involve basic research and “development” to involve applied research, our finding parallels Merk et al.^64^, who reported stronger support for research in the laboratory than in the field. Second, there was more support for regional deployment than local deployment. This seems a clear instance of a NIMBY (“not in my backyard”) mindset, where people oppose unattractive or hazardous developments near to where they live. Scholars have noted its potential to arise in public discourse about climate engineering^65^. That mindset may seem reasonable because there are potential impacts of SAI in the locality of its deployment, such as sulfur deposition and acid rain^66^. However, those effects quickly diffuse over a wider area, and the reality is that deployment anywhere has global effects. In practice, there is no local–regional distinction, and deploying SAI far away does not eliminate potential local impacts. Therefore, we question the personal utility of a NIMBY mindset to reduce exposure to the potential risks of SAI. Someone who supports only its regional deployment would still see local impacts from it.

There was also a small difference between Singapore and the United States in the levels of support for funding development. Otherwise, public support for SAI was similar. This is in contrast with Sugiyama, et al.^53^, whose results hinted at an East–West divide in opinion among developed countries. The absence of such a divide in our study may be due to non-differences in key predictors. In particular, the strongest predictors of support for funding (i.e., perceived cost and efficacy) and deployment (i.e., perceived risk and efficacy) were similar between the two countries. Among the predictors that had different mean scores between countries, none was predictive of support for funding. The perceived risk of climate change and perceived government efficacy, which were higher in Singapore, weakly predicted support for deployment. In other words, support for SAI was similar between Singapore and the United States probably because key beliefs about climate change and SAI were also similar.

The pooled regression analysis showed that support for funding was negatively related to perceived cost and unrelated to perceived risk. Also, support for deployment was negatively related to perceived risk and unrelated to perceived cost. This means that individuals oppose funding what is costly and deploying what is risky, which is intuitive and consistent with theories of public responses to perceived risks^11,18^. It makes sense that riskiness did not predict support for funding SAI research because the potential environmental and health risks do not arise until it is deployed. It makes somewhat less sense that costliness did not predict support for deployment because deployment has costs. It appears that concerns over the potential risks of SAI deployment far outweigh concerns about its financial requirements, which would explain why perceived costs were unrelated to support for deployment. In contexts where there are pronounced secondary risks, whether real or perceived, models of public opinion need to account for secondary risk perceptions^17^. Otherwise, explanations of support for deploying SAI likely will miss out on a key driver.

The multigroup regression analysis showed one difference in the prediction of support for SAI. Perceived government efficacy was a more positive predictor of support for funding in Singapore than in the United States. This finding may reflect the high degree of public trust in the Singapore government translating into support for government funding. We wish to highlight that deference to scientific authority was not a stronger predictor of support for funding in the United States than in Singapore. Although there was a difference in that direction, it was non-significant, which fails to replicate a key finding of Liang et al.^55^.

Consistent with prior research, we found that exposure to climate change information was positively related to beliefs about climate change^25^ and SAI^27^. Its relationship with perceived climate change risk is straightforward because the measures of exposure to information were specific to climate change. Its relationship with perceived SAI efficacy suggests that the participants understood SAI-relevant aspects of climate change, such as the role of solar radiation in global warming. This supports the idea that the public depends on the media and other information sources to learn about these issues^26^. Notable were the stronger effects in the United States, where exposure to information seems to play a more substantial role in opinion formation. This may be related to public views on climate change being more diverse in the United States than in Singapore^60^. Because the issue is more contentious in the United States, the public may feel more of a need to be informed and have an opinion. Alternatively, it may be that the Singapore public depends more on institutional sources, like the government and scientists, in forming their opinions. Research has shown such sources are distinct from media and interpersonal sources in the formation of climate change beliefs^25^. We do not currently have data to test that hypothesis, but it makes sense in a country where the public generally trusts a technocratic approach to policymaking^57^.

Our final analysis tested the argument that exposure to climate change information causes changes in beliefs, which cause changes in support for researching and deploying SAI. Although our cross-sectional data did not allow for tests of causation, our findings provided some evidence of a process consistent with that causal logic. In the prediction of support for both funding and deployment, there was a significant indirect effect via perceived SAI efficacy. This suggests that climate change information can trigger positive beliefs about the effectiveness of SAI as a key driver of opinion formation. The multigroup analysis showed that indirect effect was stronger in the United States than in Singapore, which further highlights potential differences in the information landscapes of those countries. Information from the media and interpersonal sources is important in both countries for opinion formation, but it seems more important in the United States.

## Conclusion

In conclusion, this study used a straightforward theoretical framework to test a parsimonious set of variables predicting public support for SAI. Those variables predicted more than two-thirds of the variance in support for funding and deploying SAI. Notably, perceived cost was negatively related to support for funding and perceived risk was negatively related to support for deployment. Finally, this study supported the idea that exposure to information is a key basis of public support for SAI because people use information to form their beliefs about it. Although our cross-sectional data precluded causal claims, the causal argument was logical and necessary for establishing the mediation model^67^. Notably, we found that exposure to information more strongly predicted beliefs in the United States than in Singapore. To understand public opinion in the United States requires careful attention to how the public accesses and uses information from media and interpersonal sources.

## Methods

Prior to data collection, we obtained ethics approval from the Institutional Review Board at Nanyang Technological University, Singapore. All methods were performed in accordance with the relevant guidelines and regulations. Data came from an online survey in English, which took place from 7 April to 9 May 2022 using Rakuten Insights online research panels. The sample included 1008 adults in Singapore (*n* = 503) and the United States (*n* = 505). The ratio of males to females was similar in Singapore (44:56) and the United States (42:58), *t*(1006) = 0.81, *p* = 0.416. The Singapore sample (*M* = 41.39, *SD* = 13.41) was younger than the United States sample (*M* = 44.16, *SD* = 15.10), *t*(1006) = 3.08, *p* = 0.002. The education level was higher in Singapore (*M* = 4.49, *SD* = 0.94) than in the United States (*M* = 4.35, *SD* = 1.12), *t*(1006) = 2.27, *p* = 0.024. Political orientation was not different between Singapore (*M* = 3.77, *SD* = 1.10) and the United States (*M* = 3.75, *SD* = 1.71), *t*(1006) = 0.22, *p* = 0.825.

### Instrument

Prior to answering questions about SAI, respondents read a short description of it to ensure they all had a basic understanding in common (see supplementary information). Given that public awareness of SAI is low^12^, including the description was reasonable and, arguably, necessary for hypotheses 7, 8, and 9 to be valid. At least one study on climate change has used similar descriptions to provide context for individuals unfamiliar with the topic^68^. We wish to highlight that our description included the benefits of SAI for reducing global warming and some of the concerns scientists have raised. Although we carefully crafted the description to reflect scientific consensus, negative information may overpower positive information^30^. This limits the ecological validity of our study.

Demographics measures included biological sex, age, and education. Education was measured on a 7-point scale from 1 (*no formal education*) to 7 (*doctoral degree*). The midpoint of 4 corresponded to an associate degree in the United States or a polytechnic degree in Singapore, which are comparable education levels. To measure political orientation, respondents rated themselves on a scale from 1 (*extremely liberal*) to 7 (*extremely conservative*). These were all single-item measures.

The survey included extant measures of communitarianism^69^ and deference to scientific authority^42^, which were control variables, and the perceived risk of climate change^70^. It included adapted measures of the perceived risk, cost, and efficacy of SAI^13,17^ and perceived government efficacy^71^. We developed face valid measures of support for funding and deployment. We modeled these variables as reflective (i.e., latent) constructs^72^, each using multiple items (see Table S1 in the supplementary information).

To measure exposure to information, participants indicated how often they use different sources for information related to climate change. The sources included television, films, radio, newspapers, magazines, books, Facebook, Twitter, YouTube, Podcasts, Reddit, Wikipedia, Friends, and Family. Response options ranged from 1 (*never*) to 4 (*frequently*). We modeled this as a formative construct^72^ by averaging the items.

### Statistical modeling

We used structural equation modeling in Mplus version 8.1 to analyze the variables in two steps. First, a measurement model estimated the latent constructs and allowed them to correlate with each other, the demographic measures, and exposure to information. Second, we estimated a mediation model of support for SAI. In that model, exposure to information predicted beliefs about climate change and SAI, which predicted support for SAI. Covariates included age, education, political orientation, and communitarianism. Using criteria by Hu and Bentler^73^, both models fit the data well (see Table S2).

There were several additional tests of statistical adequacy. First, we evaluated measurement invariance between Singapore and the United States. Using criteria by Putnick and Bornstein^74^, analyses supported an assumption of scalar invariance, meaning that the factor loadings and intercepts of the measurement items were not different between countries (see Table S2). With this level of invariance, we could validly compare latent means and regression slopes between countries^75^. Next, we assessed convergent validity of the measurement model by computing the composite reliability and the average variance extracted, which for each latent construct should exceed 0.70 and 0.50, respectively^76^. The composite reliability of deference to scientific authority was slightly below the cutoff; otherwise, there was good convergent validity (see Table S1). To assess discriminant validity, we examined the upper limit of the 95% confidence intervals of the latent variable correlations^77^. There were marginally problematic associations between the perceived risk and perceived cost of SAI (0.86) and perceived efficacy of SAI and support for deployment (0.80). The association between support for funding and deployment (0.96) was moderately problematic, suggesting a large statistical overlap between the two constructs, which is not surprising given their conceptual closeness.

### Ethics declaration

This study received ethics approval from the Institutional Review Board at Nanyang Technological University (IRB-2022–137).

### Informed consent

Informed consent was obtained from all subjects and/or their legal guardian(s).

### Supplementary Information

Supplementary Information.

## Data Availability

Data are available at https://doi.org/10.17605/OSF.IO/N5Y6B.
