# Supplementary material for: Exposure to climate change information predicts public support for solar geoengineering in Singapore and the United States
Source: Sci Rep. 2023 Nov 14;13:19874. doi: 10.1038/s41598-023-46952-w (PMC10645989; doi:10.1038/s41598-023-46952-w)
Supplement: Supplementary file 1 — Supplementary Information. [file 41598_2023_46952_MOESM1_ESM.docx]

**Description of SAI**

The description below was drafted by the lead author and vetted by the second author, an expert on SAI. It appeared in the survey after the demographics and before the questions on beliefs about and support for SAI.

*Solar geoengineering makes changes to the air or surface of the Earth to reduce the amount of sunlight that reaches the ground. One type of solar geoengineering that scientists are exploring is called stratospheric aerosol injection. Naturally, erupting volcanoes release gases into the upper atmosphere that block sunlight from reaching the Earth’s surface. This can cool the planet. Based on that effect, scientists have proposed using high-altitude aircraft or balloons to release special gases into the atmosphere to help make the planet cooler. But there may be some problems with this solution. Some scientists think the release of the gases can harm the ozone layer. The gases may also harm ecosystems and agriculture. Finally, the gases can drift down to the lower atmosphere, which is where clouds form, and cause more acid rain.*

**Figure S1: Levels of support for SAI in Singapore and the United States**


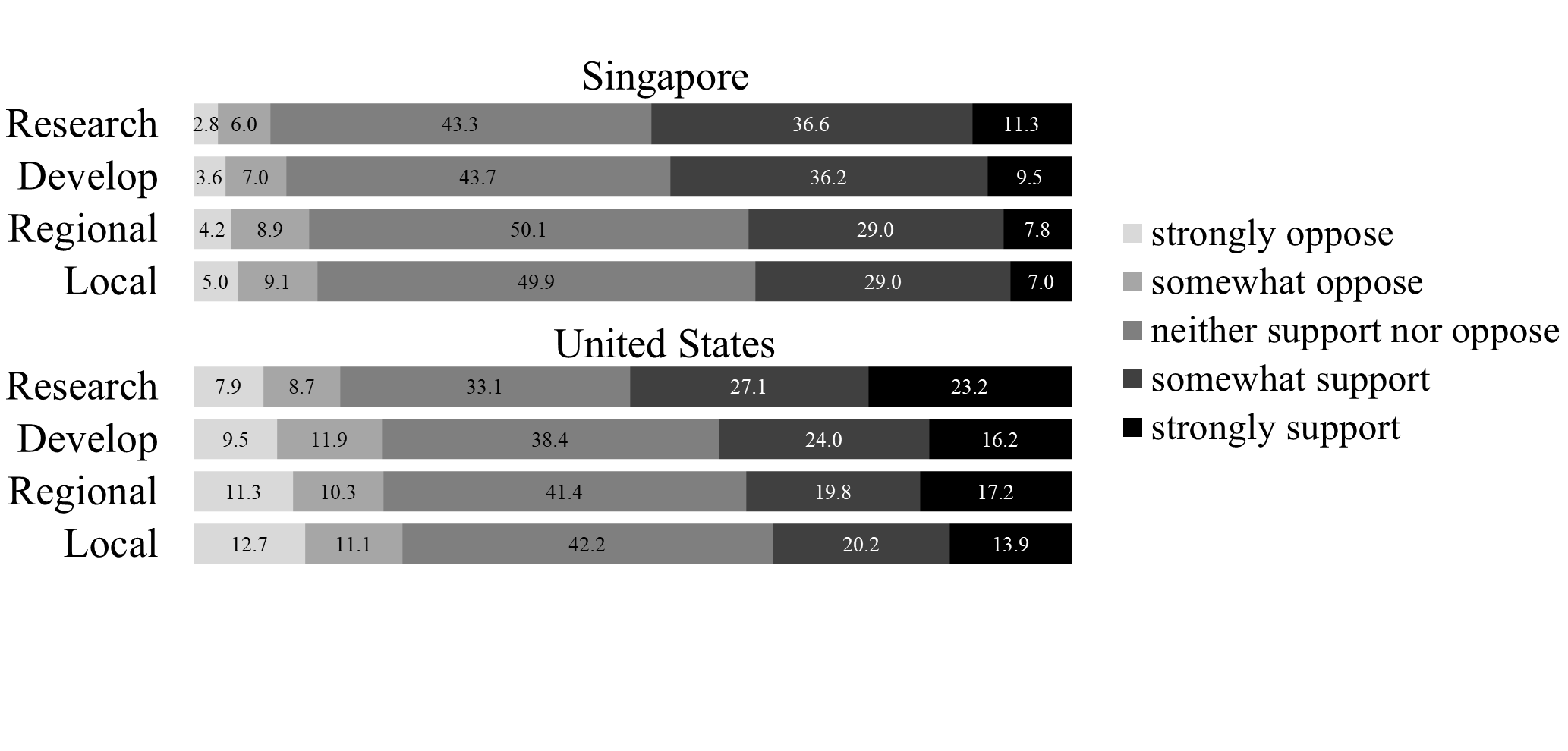


*Note*. Research = support for funding research. Develop = support for funding development. Regional = support for regional deployment. Local = support for local deployment. Numbers in the shaded areas represent the percentage of each response option. Each bar adds up to 100% with rounding error.

**Table S1: Item wording and descriptive statistics (pooled sample)**

| Latent constructs and measurement items | *M* | *SD* | λ |
| --- | --- | --- | --- |
| **Support for funding SAI (CR = 0.85; AVE = 0.73)** |  |  |  |
| Government funding of research on stratospheric aerosol injection^1^ | 3.48 | 1.03 | 0.82 |
| Government funding for developing stratospheric aerosol injection^1^ | 3.33 | 1.03 | 0.89 |
| **Support for deploying SAI (CR = 0.87; AVE = 0.77)** |  |  |  |
| The use of stratospheric aerosol injection in [Southeast Asia / the United States]^1^ | 3.24 | 1.04 | 0.88 |
| The use of stratospheric aerosol injection in [Singapore / my city or town]^1^ | 3.18 | 1.04 | 0.88 |
| **Communitarianism (CR = 0.76; AVE = 0.62)** |  |  |  |
| The government should do more to advance society's goals, even if that means limiting the freedom and choices of individuals.^2^ | 3.11 | 1.12 | 0.81 |
| Government should put limits on the choices individuals can make so they don't get in the way of what's good for society.^2^ | 3.02 | 1.12 | 0.76 |
| **Scientific deference (CR = 0.67; AVE = 0.51)** |  |  |  |
| Scientists should move ahead with research even if it displeases some people.^2^ | 3.57 | 1.00 | 0.74 |
| Our leaders should use technology to solve problems in society.^2^ | 3.51 | 0.98 | 0.69 |
| **Perceived risk of CC (CR = 0.81; AVE = 0.58)** |  |  |  |
| How much do you think climate change will harm you personally?^3^ | 3.02 | 0.84 | 0.82 |
| How much do you think climate change will harm future generations of people?^3^ | 3.43 | 0.81 | 0.74 |
| I feel worried about climate change.^2^ | 3.86 | 1.10 | 0.72 |
| **Perceived risk of SAI (CR = 0.87; AVE = 0.62)** |  |  |  |
| Stratospheric aerosol injection could have considerable negative consequences.^2^ | 3.50 | 0.87 | 0.77 |
| The negative effects of stratospheric aerosol injection could be severe.^2^ | 3.48 | 0.89 | 0.81 |
| There is a serious risk of stratospheric aerosol injection harming people.^2^ | 3.47 | 0.89 | 0.77 |
| I feel worried about stratospheric aerosol injection.^2^ | 3.57 | 0.90 | 0.79 |
| **Perceived cost of SAI (CR = 0.72; AVE = 0.56)** |  |  |  |
| Using stratospheric aerosol injection to fight climate change will be expensive for taxpayers.^2^ | 3.56 | 0.89 | 0.77 |
| Developing stratospheric aerosol injection to fight climate change will take money from more important projects.^2^ | 3.37 | 0.93 | 0.73 |
| **Perceived efficacy of SAI (CR = 0.85; AVE = 0.66)** |  |  |  |
| Stratospheric aerosol injection can be effective in reducing the threat of climate change.^2^ | 3.37 | 0.90 | 0.80 |
| Stratospheric aerosol injection can help prevent the risks of climate change.^2^ | 3.37 | 0.91 | 0.83 |
| Stratospheric aerosol injection is a good solution to climate change.^2^ | 3.22 | 0.93 | 0.81 |
| **Perceived govt efficacy (CR = 0.90; AVE = 0.75)** |  |  |  |
| How much confidence do you have in the government's ability to respond to climate change?^3^ | 2.72 | 1.15 | 0.87 |
| How effective do you think the government's actions are in the fight against climate change?^4^ | 2.68 | 1.12 | 0.90 |
| How capable do you think the government is to respond to climate change?^5^ | 2.86 | 1.16 | 0.82 |

*Note*. CC = climate change. SAI = stratospheric aerosol injection. *M* = mean. *SD* = standard deviation. λ = standardized factor loading. CR = composite reliability (> 0.70 is desirable), indicating the consistency of measurement. AVE = average variance extracted (> 0.50 is desirable), indicating how well the latent construct explains its measurement items, which is related to the consistency of measurement. ^1^Response options ranged from 1 (*strongly oppose*) to 5 (*strongly support*).  ^2^Response options ranged from 1 (*strongly disagree*) to 5 (*strongly agree*). ^3^Response options ranged from 1 (*not at all*) to 5 (a great deal). ^4^Response options ranged from 1 (*not effective at all*) to 5 (*extremely effective*). ^5^Response options ranged from 1 (*not capable at all*) to 5 (*extremely capable*).

**Table S2: Linear regression of support for SAI funding in Singapore (*R*^2^ = 0.55) and the United States (*R*^2^ = 0.80)**

|  |  | β [95% CI] | |  |
| --- | --- | --- | --- | --- |
| Predictors |  | Singapore | United States | χ^2^(1) |
| Sex |  | -0.025 [-0.114, 0.064] | 0.032 [-0.051, 0.115] | 1.09 |
| Age |  | 0.006 [-0.083, 0.095] | 0.018 [-0.083, 0.119] | 0.05 |
| Education |  | 0.007 [-0.083, 0.097] | 0.009 [-0.075, 0.093] | 0.00 |
| Political orientation |  | -0.031 [-0.121, 0.059] | 0.046 [-0.035, 0.127] | 1.85 |
| Communitarianism |  | -0.069 [-0.259, 0.121] | -0.033 [-0.239, 0.174] | 0.22 |
| Perceived CC risk |  | 0.081 [-0.046, 0.208] | -0.025 [-0.177, 0.127] | 1.75 |
| Perceived SAI efficacy | 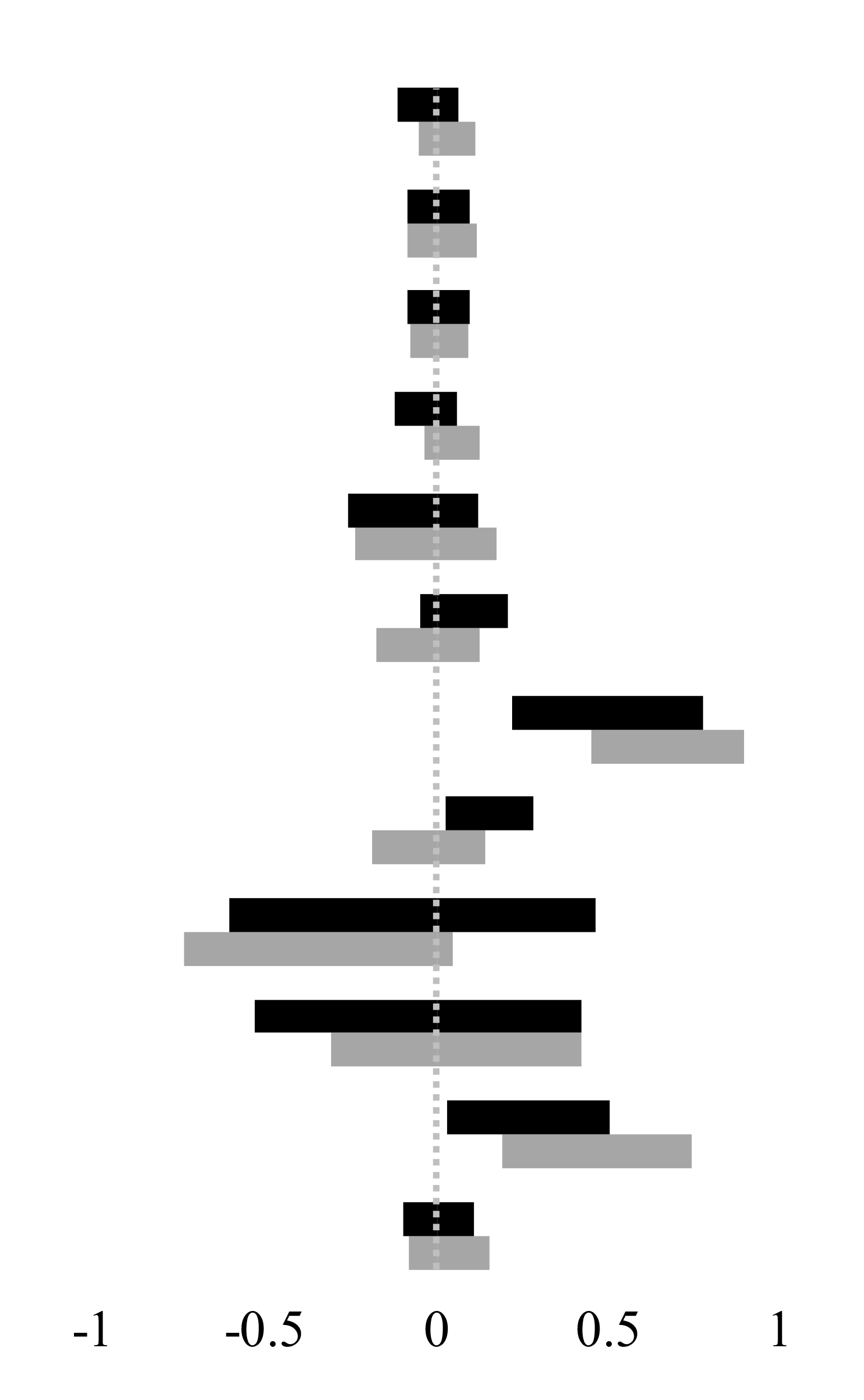 | 0.497 [0.219, 0.776] | 0.672 [0.450, 0.895] | 1.43 |
| Perceived govt efficacy |  | 0.155 [0.026, 0.284] | -0.023 [-0.187, 0.140] | 4.48* |
| Perceived SAI cost |  | -0.071 [-0.605, 0.463] | -0.345 [-0.736, 0.047] | 1.93 |
| Perceived SAI risk |  | -0.053 [-0.530, 0.423] | 0.058 [-0.309, 0.424] | 0.46 |
| Scientific deference |  | 0.267 [0.029, 0.505] | 0.467 [0.192, 0.743] | 2.30 |
| Exposure to information |  | 0.007 [-0.096, 0.110] | 0.036 [-0.082, 0.154] | 0.17 |

*Note*. CC = climate change. SAI = stratospheric aerosol injection. Bars show the 95% confidence intervals (CI) of the standardized regression paths (β) in Singapore (black) and the United States (gray). Confidence intervals were estimated with 5,000 bias-corrected bootstrap samples. χ^2^(1) is the chi-square test with one degree of freedom comparing each regression path between countries. **p* < .05.

**Table S3: Linear regression of support for SAI deployment in Singapore (*R*^2^ = 0.68) and the United States (*R*^2^ = 0.71)**

|  |  | β [95% CI] | |  |
| --- | --- | --- | --- | --- |
| Predictors |  | Singapore | United States | χ^2^(1) |
| Sex |  | -0.043 [-0.126, 0.041] | -0.014 [-0.091, 0.064] | 0.16 |
| Age |  | 0.020 [-0.066, 0.107] | 0.004 [-0.092, 0.100] | 0.06 |
| Education |  | 0.039 [-0.041, 0.120] | -0.032 [-0.111, 0.046] | 1.80 |
| Political orientation |  | 0.006 [-0.083, 0.096] | 0.058 [-0.018, 0.133] | 0.80 |
| Communitarianism |  | -0.042 [-0.210, 0.126] | 0.110 [-0.062, 0.283] | 2.16 |
| Perceived CC risk |  | 0.094 [-0.025, 0.213] | 0.050 [-0.087, 0.187] | 0.47 |
| Perceived SAI efficacy |  | 0.552 [0.305, 0.799] | 0.590 [0.383, 0.797] | 0.03 |
| Perceived govt efficacy | 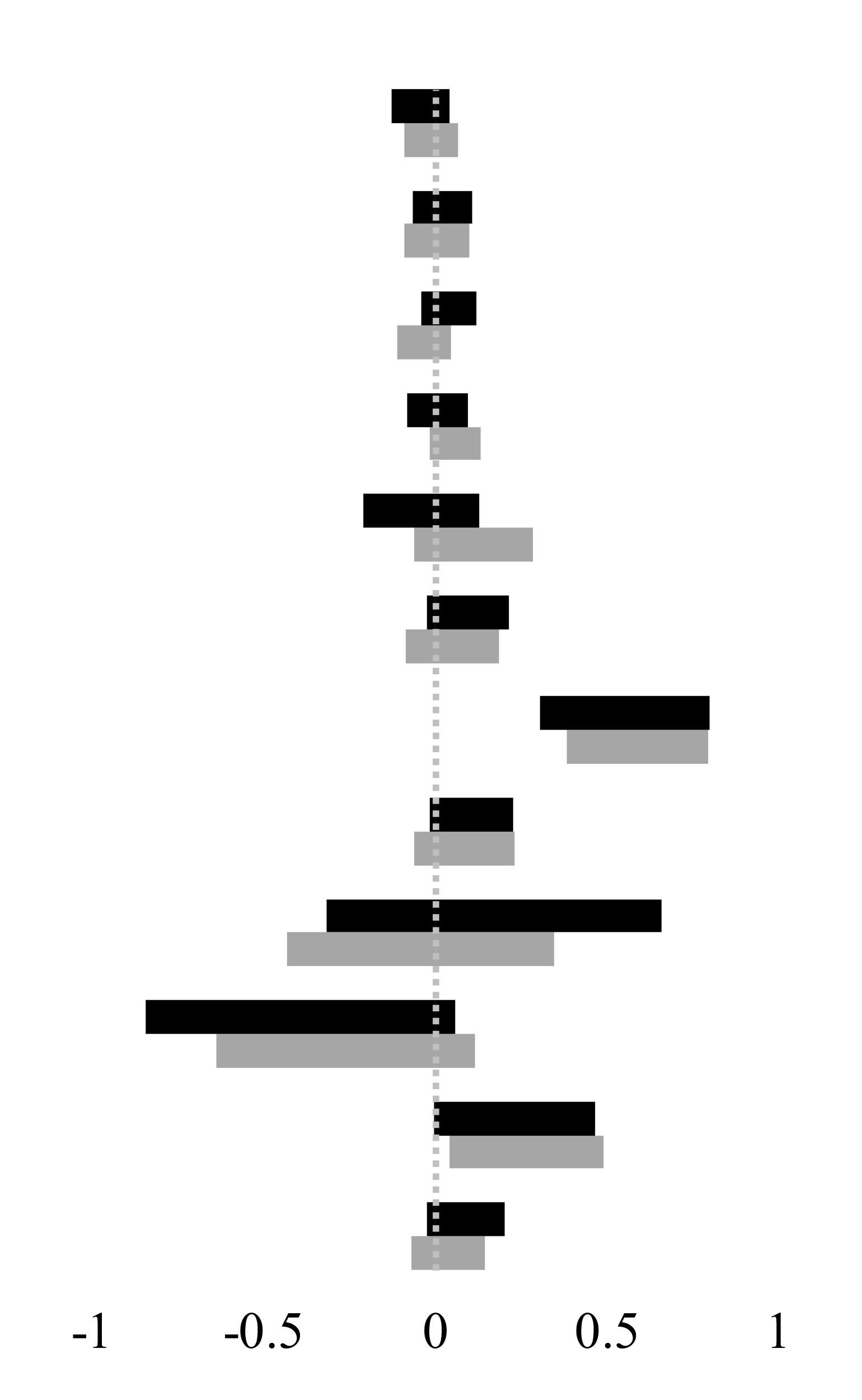 | 0.106 [-0.015, 0.226] | 0.085 [-0.060, 0.230] | 0.07 |
| Perceived SAI cost |  | 0.171 [-0.317, 0.660] | -0.041 [-0.431, 0.348] | 1.73 |
| Perceived SAI risk |  | -0.393 [-0.843, 0.057] | -0.262 [-0.638, 0.114] | 0.93 |
| Scientific deference |  | 0.233 [-0.001, 0.466] | 0.266 [0.042, 0.489] | 0.09 |
| Exposure to information |  | 0.087 [-0.026, 0.200] | 0.038 [-0.068, 0.143] | 0.41 |

*Note*. CC = climate change. SAI = stratospheric aerosol injection. Bars show the 95% confidence intervals (CI) of the standardized regression paths (β) in Singapore (black) and the United States (gray). Confidence intervals were estimated with 5,000 bias-corrected bootstrap samples. χ^2^(1) is the chi-square test with one degree of freedom comparing each regression path between countries, none of which was significant.

**Table S4: Effects of exposure to information on beliefs in Singapore and the United States**

|  |  | β [95% CI] | |  |
| --- | --- | --- | --- | --- |
| Outcome |  | Singapore | United States | χ^2^(1) |
| Perceived CC risk |  | 0.235 [0.122, 0.349] | 0.371 [0.262, 0.480] | 5.79* |
| Perceived SAI efficacy | 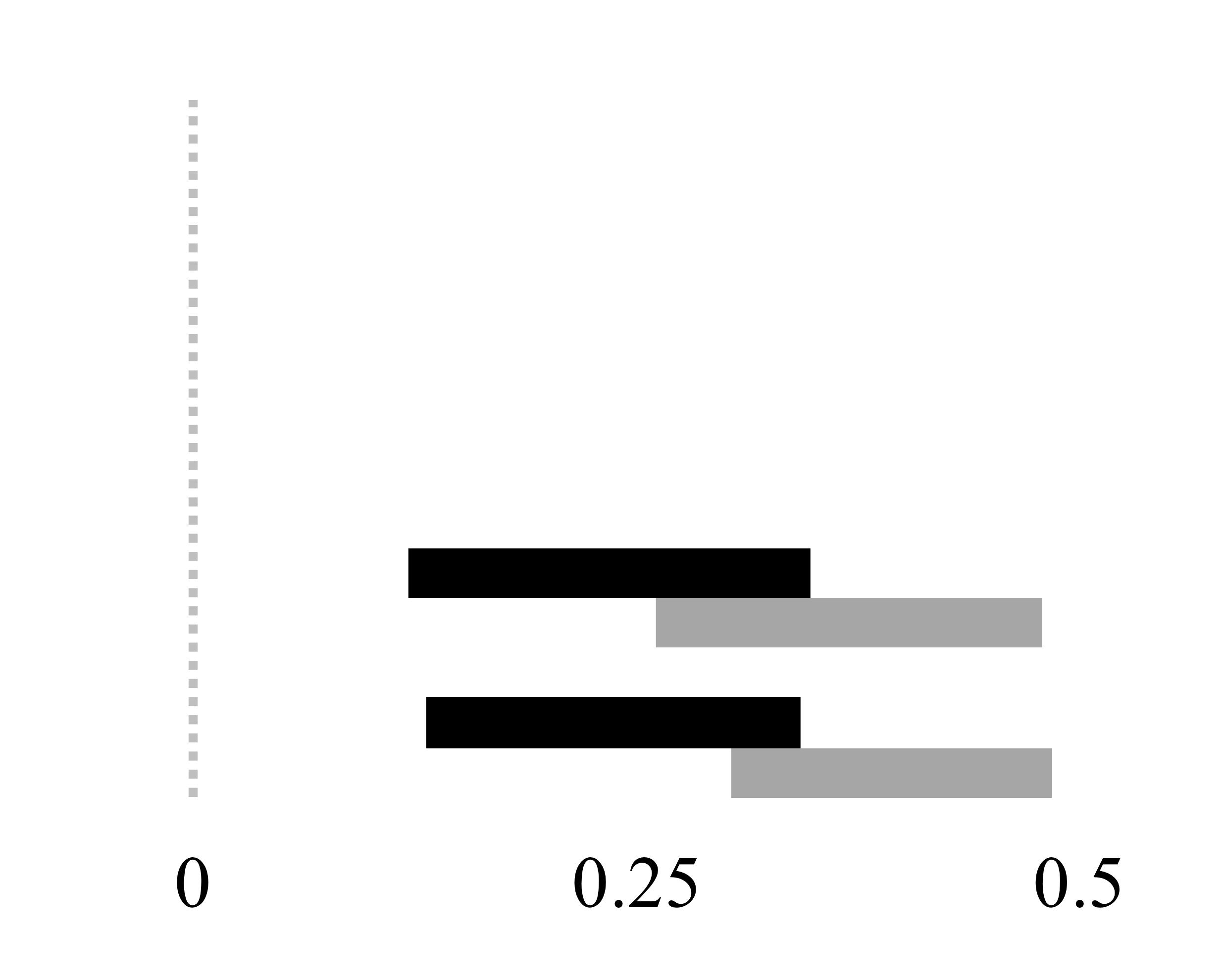 | 0.237 [0.132, 0.343] | 0.394 [0.304, 0.485] | 7.15** |

*Note*. CC = climate change. SAI = stratospheric aerosol injection. Bars show the 95% confidence intervals (CI) of the standardized regression paths (β) in Singapore (black) and the United States (gray). Confidence intervals were estimated with 5,000 bias-corrected bootstrap samples. χ^2^(1) is the chi-square test with one degree of freedom comparing each regression path between countries. **p* < .05. ***p* < .01.
